# Factors Affecting Compliance and Visual Outcomes in Patients Receiving Intravitreal Bevacizumab Injections

**DOI:** 10.7759/cureus.66760

**Published:** 2024-08-13

**Authors:** Ozukhil Radhakrishnan, Tushar Agrawal, Nilesh Giri, Shreya Gandhi, Khushboo Goyal

**Affiliations:** 1 Ophthalmology, Dr. D. Y. Patil Medical College, Hospital and Research Centre, Dr. D. Y. Patil Vidyapeeth (Deemed to be University), Pune, IND

**Keywords:** branch retinal vein occlusion, central retinal vein occlusion (crvo), n-armd, diabetic retinopathy, bevacizumab therapy, anti-vegf treatment

## Abstract

Background

Vascular endothelial growth factor (VEGF) is a powerful mitogen for endothelial cells that promotes migration, proliferation, and tube formation necessary for the angiogenic development of new blood vessels. When VEGF increases significantly, it causes pathological angiogenesis and increased vascular permeability in eye conditions such as diabetic retinopathy (DR), age-related macular degeneration (AMD), and retinal vein occlusion (RVO). These disorders have become important global sources of morbidity and have a substantial financial impact not only on the medical community but also on the patients. Therefore, this study aims to determine the success rates of intravitreal bevacizumab therapy and the visual outcomes which may be increased by determining the factors affecting patient compliance and raising awareness about DR, neovascular AMD, and RVO among patients and studying the factors responsible for non-compliance to treatment.

Methodology

This experimental pre-post study was conducted in the ophthalmology department at a tertiary care hospital and research center in western Maharashtra from September 2022 to June 2024. A total of 150 eyes of 150 patients who were diagnosed cases of DR, neovascular AMD, and non-ischemic RVO were included in the study. Written informed consent was obtained from each patient. Data were entered in Microsoft Excel (Microsoft Corp., Redmond, WA, USA) and statistical analysis was done using SPSS 27.0 software (IBM Corp., Armonk, NY, USA). The chi-square test was employed to check the association between categorical variables. Pearson’s correlation test was used, and p-values <0.05 were considered significant.

Results

The compliance rate in our study was 79.3% (113 individuals). In our study, 58% (87 individuals) were male while 42% (63 individuals) were females. Most patients were from urban areas 74.7% (112 individuals). Among the study participants, DR patients constituted 48.6% (73 individuals), while neovascular AMD and RVO were seen in 32% (48 individuals) and 19.4% (29 individuals), with 62% (93 individuals) being diabetic and 64.7% (97 individuals) being hypertensive. In our study, 92% (138 individuals) were willing to take treatment, with 88.7% (133 individuals) worried about their visual outcomes and 66% (99 individuals) afraid of getting injected. Appointments posed a financial burden to 30.7% (46 individuals) of patients, with 55.3% (83 individuals) having transportation issues. Overall, 18.7% (28 individuals) of patients had missed appointments between 14 and 90 days while 30.7% (46 individuals) had missed their appointments by 90 and 365 days. Younger patients with a shorter duration of diabetes had higher compliance rates. Post-injection, there was an overall significant improvement in vision as well as a reduction in the central subfield macular thickness, volume cube, and thickness average cube.

Conclusions

In the present study, four-fifths of the patients were compliant with treatment, and visual improvement was significant. In addition, there was a significant reduction in the macular thickness after the treatment. One of the factors for non-compliance included in our study was the need for follow-up. Younger patients and those with a shorter duration of diabetes had significantly higher compliance. We recommend further studies should be conducted to compare the effectiveness with the control group in randomized control trials.

## Introduction

Vascular endothelial growth factor (VEGF) is a dimeric glycoprotein with a molecular weight of around 40 kDa. It is a powerful mitogen for endothelial cells that promotes migration, proliferation, and tube formation, all of which are necessary for the angiogenic development of new blood vessels. It is necessary for angiogenesis to occur throughout development. The loss of a single allele stops angiogenesis and results in embryonic mortality [1].

When it comes to significant eye conditions such as diabetic retinopathy (DR) and age-related macular degeneration (AMD), the VEGF family regulates pathological angiogenesis and increased vascular permeability. According to the literature, increased microvascular permeability and angiogenesis are caused by the overexpression of VEGFs and their receptors, VEGFR-1, VEGFR-2, and VEGFR-3. Current research indicates that VEGFR-1 functions as a functional receptor for VEGF-A and placenta growth factor in vascular smooth muscle cells and pericytes in vivo, as opposed to endothelial cells. This strongly suggests that pericytes are involved in the early stages of angiogenesis. The cellular distribution of VEGFR-1, VEGFR-2, and VEGFR-3 indicates that the VEGF family has various distinct roles in the normal retina, including both neuronal and retinal vascular activities. Moreover, it is probable that the retinal pigment epithelium (RPE) and other epithelia release VEGFs, which transmit paracrine vascular survival signals for neighboring endothelia. Derailment of this paracrine relationship and RPE upregulation of VEGF-A in the choroid might explain the pathophysiology of AMD-related subretinal neovascularization. However, as is now being done in several clinical studies for AMD and DR, therapeutic VEGF suppression may jeopardize this paracrine relationship as well as other physiological roles of VEGFs [2].

In recent years, intravitreal injections of VEGF inhibitors have replaced other methods of treatment as the gold standard for many retinal and chorioretinal illnesses. Retinal disorders, such as diabetic macular edema, AMD, and retinal vein occlusion (RVO), are important global sources of morbidity and have a substantial financial impact on both the medical community and patients. First, pegaptanib sodium was used to treat neovascular age-related macular degeneration (nAMD). Later, these treatments were expanded to treat diabetic macular edema, macular edema caused by retinal vein blockage, and choroidal neovascularization [3].

A humanized monoclonal antibody directed against VEGF-A is called bevacizumab [4]. Recent research has shown that intravitreal injection of bevacizumab can help reduce fibrovascular proliferation and vascular permeability in cases of choroidal neovascularization secondary to AMD, retinal neovascularization secondary to proliferative DR, and macular edema secondary to central vein occlusion [5,6].

For achieving expected healthcare benefits, adherence to any treatment is very important. Hence, patients are expected to be compliant and follow up regularly to receive their next scheduled dosage of intravitreal anti-VEGF. However, poor compliance has been observed. Factors that lead to non-compliance to anti-VEGF therapy include whether they are educated about the need for injections, any comorbidities, willingness to take treatment, treatment disbelief, family support, financial burden, transportation issues, etc. [7].

This study aims to determine the compliance and success rates of bevacizumab therapy as well as the visual outcome which may be increased by determining the factors affecting patient compliance and raising awareness about DR, nAMD, and RVO among patients and studying the factors responsible for non-compliance to treatment. At present, there are few studies on the compliance factors and success rates of bevacizumab therapy from western Maharashtra, India.

## Materials and methods

Study design and setting

This experimental pre-post study was conducted in the ophthalmology department at a tertiary care hospital and research center in western Maharashtra from September 2022 to June 2024. A total of 150 eyes of 150 patients who were diagnosed cases of DR, nAMD, and non-ischemic RVO were included in the study, with each participant undergoing a thorough clinical assessment and investigation. The study received approval from the Institutional Ethics Committee of Dr. D. Y. Patil Medical College, Hospital and Research Centre, Pune (approval number: IESC/PGS/2022/109).

Inclusion criteria

All patients who were diagnosed with cases of nAMD, DR, and non-ischemic RVO in the age group above 18 years with a follow-up for at least six months after the first injection at a tertiary care center in western Maharashtra were included in the study.

Exclusion criteria

Patients with corneal pathology such as corneal scars, dense cataracts, or vitreous hemorrhage preventing good visualization of the fundus were excluded from the study. We also excluded patients with non-ischemic RVO, those less than 18 years of age, those with a history of previous vitreoretinal surgery or laser procedures or any other intraocular injection received in either eye, and those not willing to undergo the procedure or not consenting to the study.

Sample size

Considering the mean and standard deviation (SD) of pre and post-central macular thickness 492.8 ± 167.2 μm and 379 ± 104 μm, respectively, from a previous study and with a 95% confidence interval (CI) and 80% power, the minimum sample size calculated was 48. However, we considered a sample size of 150 in our study. The software used for calculating the sample size was WinPepi version 11.38 [8].

Patient details were obtained initially, i.e., age, sex, and diabetic or hypertensive profile. The factors that affect compliance, i.e., occupation, residence, employment status, financial status, number of injections, details of follow-up, and missed appointments were enquired. After a detailed history, each patient went through a baseline examination which included best-corrected visual acuity correction measurement with Snellen/ETDRS charts and ophthalmic examination, including slit lamp bio-microscopy, intraocular pressure measurement on Goldmann applanation tonometer before and after injection and dilated fundus examination with +90 diopter lens, and indirect ophthalmoscopy. All patients had baseline central subfield macular thickness analyzed by optical coherence tomography (OCT) and fundus photography. Fundus fluorescein angiography was done wherever required. Patients were followed up in the outpatient department (OPD) and the reasons for non-compliance were noted using an oral questionnaire (developed by Dr. Tushar Agrawal). Patients were counseled to choose only one reason. No follow-up between 14 days to 90 days was considered a missed appointment, no follow-up between 90 and 365 was considered a break-off, and no follow-up visit for more than 365 days was considered as lost to follow-up. Reasons for break-off or loss to follow-up were noted if available. These injections are usually recommended once a month depending on the OCT scan and fundus photography. The injection site was prepared using topical proparacaine 0.5% and povidone-iodine 5.0%. Povidone-iodine-soaked swab sticks were used to sterilize the eyelids and surrounding tissue. The injection site was in the inferotemporal quadrant due to its ease of accessibility. Using a caliper, distance was measured which was 4.0 mm in phakic eyes to prevent inadvertent trauma of the crystalline lens and 3.5 mm in aphakia and pseudophakia eyes which was done in the operation theater.

Consent

Written informed consent was obtained as per the Declaration of Helsinki from each patient. In the study, we recruited 150 patients and explained the procedure and purpose to them. Informed consent was obtained from all patients.

Statistical analysis

Data was entered in MS Excel (Microsoft Corp., Redmond, WA, USA), and statistical analysis was done using SPSS 27.0 software (IBM Corp., Armonk, NY, USA). As the continuous variables showed skewed distribution, we used the Mann-Whitney test and the Wilcoxon signed-rank test to test the significance between continuous and categorical variables. The chi-square test was employed to check the association between categorical variables. Pearson’s correlation test was used, and p-values <0.05 were considered significant.

## Results

A total of 150 eyes that underwent intravitreal injection of bevacizumab (1.25 mg/0.05 mL) who met the inclusion criteria underwent ocular evaluation in the ophthalmology OPD.

In our study, of the 150 total participants, 58% were males and 42% were females. The most common occupations of the individuals enrolled in our study were businessmen (28%) while 24.7% were housewives. Most patients in our study were graduates (63.3%), while 20.7% were uneducated and 16% were 10th pass. Moreover, the majority of the individuals were retired (50%). Most patients in our study hailed from urban backgrounds (74.7%), while 25.3% were from rural areas. Overall, 69.3% of the patients were smokers while 30.7% of the individuals were non-smokers.

The mean age of the patients studied was 59.51 years. The mean duration of diabetes was 9.79 years, the mean HbA1C level was 8.1, the mean duration of hypertension was 4.19 years, and the mean pre-intravitreal injection central subfield macular thickness and post-intravitreal injection central subfield macular thickness were 393.87 and 301.42, respectively. The mean pre-intravitreal injection volume cube and post-intravitreal injection volume cube were 11.33 mm^3^ and 10.27 mm^3^, respectively. The mean pre-intravitreal injection thickness and post-intravitreal injection thickness were 313.2 mm^3^ and 285.59 mm^3^, respectively (Table 1).

**Table 1:** Descriptive statistics of demographic and clinical parameters in the study population. DM = diabetes mellitus; SD = standard deviation

Parameters	Mean ± SD
Age	59.51 ± 10.08
Duration of DM	9.79 ± 8.58
HBA1c levels	8.10 ± 1.42
Duration of hypertension	4.19 ± 4.71
Pre-intravitreal injection central subfield macular thickness (μm)	393.87 ± 195.86
Pre-intravitreal injection volume cube (mm^3^)	11.33 ± 3.64
Pre-intravitreal injection thickness average cube (μm)	313.20 ± 98.75
Post-intravitreal injection central subfield macular thickness (μm)	301.42 ± 130.18
Post-intravitreal injection volume cube (mm^3^)	10.27 ± 3.95
Post-intravitreal injection thickness average cube (μm)	285.59 ± 112.27

In our study, DR patients constituted 48.6%, while nAMD was seen in 32% and RVO in 19.3% of patients (Table 2).

**Table 2:** Diagnosis of individuals in the study population. NPDR = non-proliferative diabetic retinopathy; PDR = proliferative diabetic retinopathy; nAMD = neovascular age-related macular degeneration; RVO = retinal vein occlusion

Diagnosis	Frequency (Percent)
Mild to severe NPDR	38 (25.3%)
PDR	35 (23.3%)
nAMD	48 (32%)
RVO	29 (19.4%)
Total	150 (100%)

In our study, diabetes mellitus was prevalent among 62% of the patients, and among the diabetics, 53.8% were on glitazones. Hypertension was prevalent among 64.7% of the patients, and among these individuals, 85.6% were taking drugs for the management of hypertension and 45.3% had a positive family history of diabetes and hypertension (Table 3).

**Table 3:** Individuals with diabetes mellitus, hypertension, and a family history of both in the study population.

Systemic illness		Frequency (Percent)
Diabetes mellitus	Yes	93 (62%)
	No	57 (38%)
	Total	150 (100%)
Hypertension	Yes	97 (64.7%)
	No	53 (35.3%)
	Total	150 (100%)
Family history of diabetes mellitus and hypertension	Yes	68 (45.3%)
	No	82 (54.7%)
	Total	150 (100%)

In our study, the compliance rate for the treatment was 79.3% respectively (Table 4).

**Table 4:** Treatment compliance in the study population.

Compliant with treatment	Frequency (Percent)
Yes	119 (79.3%)
No	31 (20.7%)
Total	150 (100%)

A questionnaire was designed to study factors affecting compliance among patients receiving intravitreal bevacizumab injections (Table 5).

**Table 5:** Questionnaire designed to study the factors affecting compliance of patients receiving intravitreal bevacizumab injections. Table credit: Tushar Agrawal.

Questionnaire	Yes	No
Do you have any comorbidities?	76	74
Are you willing to get treatment for your eye disease?	138	12
Are you well informed about your eye disease?	150	0
Are you worried about your visual outcome?	133	17
Are you afraid of the side effects of the injection?	80	70
Do you know the necessity of taking these injections?	133	17
Are you afraid of getting injected?	99	51
Any disbelief in the benefit of the treatment?	11	139
Are you experiencing a lack of support from the family for this treatment?	20	130
Did you get relocated from your residential address?	34	116
Do the appointments pose a financial burden to you and your family?	46	104
Are you having difficulty taking time out from your job?	32	118
Are you facing transportation issues?	83	67
Are you going to some other hospital for the same treatment?	24	126
Are you going to some other hospital for the comorbid disease treatment?	70	80
Are you entitled to any health schemes?	150	0

Overall, 50.7% (76 individuals) had other comorbidities, 92% (138 individuals) were willing to get treated, all 150 patients were well informed about their disease and its treatment, 88.7% (133 individuals) expressed that they were worried about their visual outcome, 53.3% (80 individuals) were afraid about the side effects of the injections, 88.7% (133 individuals) were aware of the necessity for taking these injections, 66.7% (99 individuals) were afraid of getting injected, 7.3% (11 individuals) expressed disbelief of the treatment, 13.3% (20 individuals) experienced lack of support from the family for treatment, 22.7% (34 individuals) were relocated from their residential address, 30.7%(46 individuals) expressed that appointments pose a financial burden to them and their family. Further, 21.3% (32 individuals) expressed difficulty taking out time from their job, 55.3% (83 individuals) reported transportation issues, 16% (24 individuals) were going to some other hospital for the same treatment, and 46.7% (70 individuals) were attending some other hospital for their comorbid disease treatment. All patients were enrolled under a health insurance scheme.

The majority of the patients took at least one anti-VEGF injection (65.3%) during the study period (Table 6).

**Table 6:** Number of anti-VEGF injections taken during the study period. VEGF = vascular endothelial growth factor

Number of injections taken	Frequency (Percent)
1	98 (65.3%)
2	30 (20%)
3	14 (9.3%)
4	5 (3.3%)
5	3 (2%)
Total	150 (100%)

**Figure 1:**
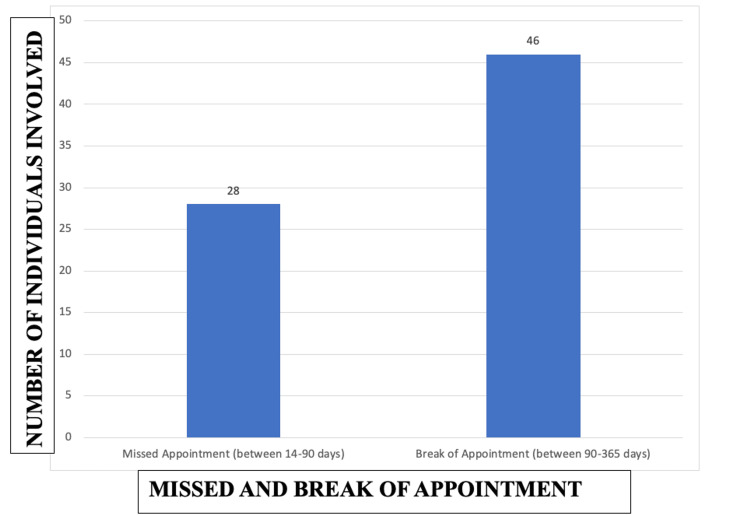
Missed appointments and appointment breaks in the study population.

Overall, 18.7% (28 individuals) had missed their appointments by 14 to 90 days while 30.7% (46 individuals) had missed their appointments by 90 to 365 days (Figure 1).

According to the chi-square test, there was no association between the diagnosis and treatment compliance, with a p-value of 0.073 (Table 7).

**Table 7:** Treatment compliance as per diagnosis in the study population. Chi-square test. NPDR = non-proliferative diabetic retinopathy; PDR = proliferative diabetic retinopathy; nAMD = neovascular age-related macular degeneration; RVO = retinal vein occlusion

Diagnosis		Compliant with treatment		P-value
		Yes	No	
Mild to severe NPDR	Frequency (percent)	25 (65.8%)	13 (34.2%)	0.073
PDR	Frequency (percent)	30 (85.7%)	5 (14.3%)	
nAMD	Frequency (percent)	38 (79.2%)	10 (20.8%)	
RVO	Frequency (percent)	26 (89.7%)	3 (10.3%)	
Total	Frequency (percent)	119 (79.3%)	31 (20.7%)	

Furthermore, in our study, through univariate analysis by the chi-square test, there was no association between gender and treatment compliance (p = 0.223); no association between occupation and treatment compliance (p = 0.662); no association between education and treatment compliance (p = 0.329); no association between employment status and the treatment compliance (p = 0.717); no association between urban/rural status and the treatment compliance (p = 0.946); no association between financial status and the treatment compliance (p = 0.692); no association between smoking and treatment compliance (p = 0.125); no association between the presence of diabetes mellitus and treatment compliance (p = 0.116); and no association between patient being a hypertensive and treatment compliance (p = 0.688).

Through the Mann-Whitney test, younger patients (p = 0.032) with a shorter duration of diabetes (p = 0.009) had significantly higher compliance rates (Table 8).

**Table 8:** Compliance with treatment in comparison to age, duration of diabetes, HbA1c levels, and duration of diabetes. Mann-Whitney test. Out of 150 patients, 93 patients were diabetic and 97 patients were hypertensive. Hence, compliance with treatment was studied according to this data. DM = diabetes mellitus

Parameters	Compliance with treatment	Number of individuals	Mean rank	Sum of ranks	P-value
Age	Yes	119	71.62	8,522.50	0.032
	No	31	90.40	2,802.50	
	Total	150			
Duration of DM	Yes	70	42.81	2,997.00	0.009
	No	23	59.74	1,374.00	
	Total	93			
HBA1c levels	Yes	70	47.59	3,331.50	0.711
	No	23	45.20	1,039.50	
	Total	93			
Duration of Hypertension	Yes	76	51.17	3,889.00	0.145
	No	21	41.14	864.00	
	Total	97			

**Figure 2:**
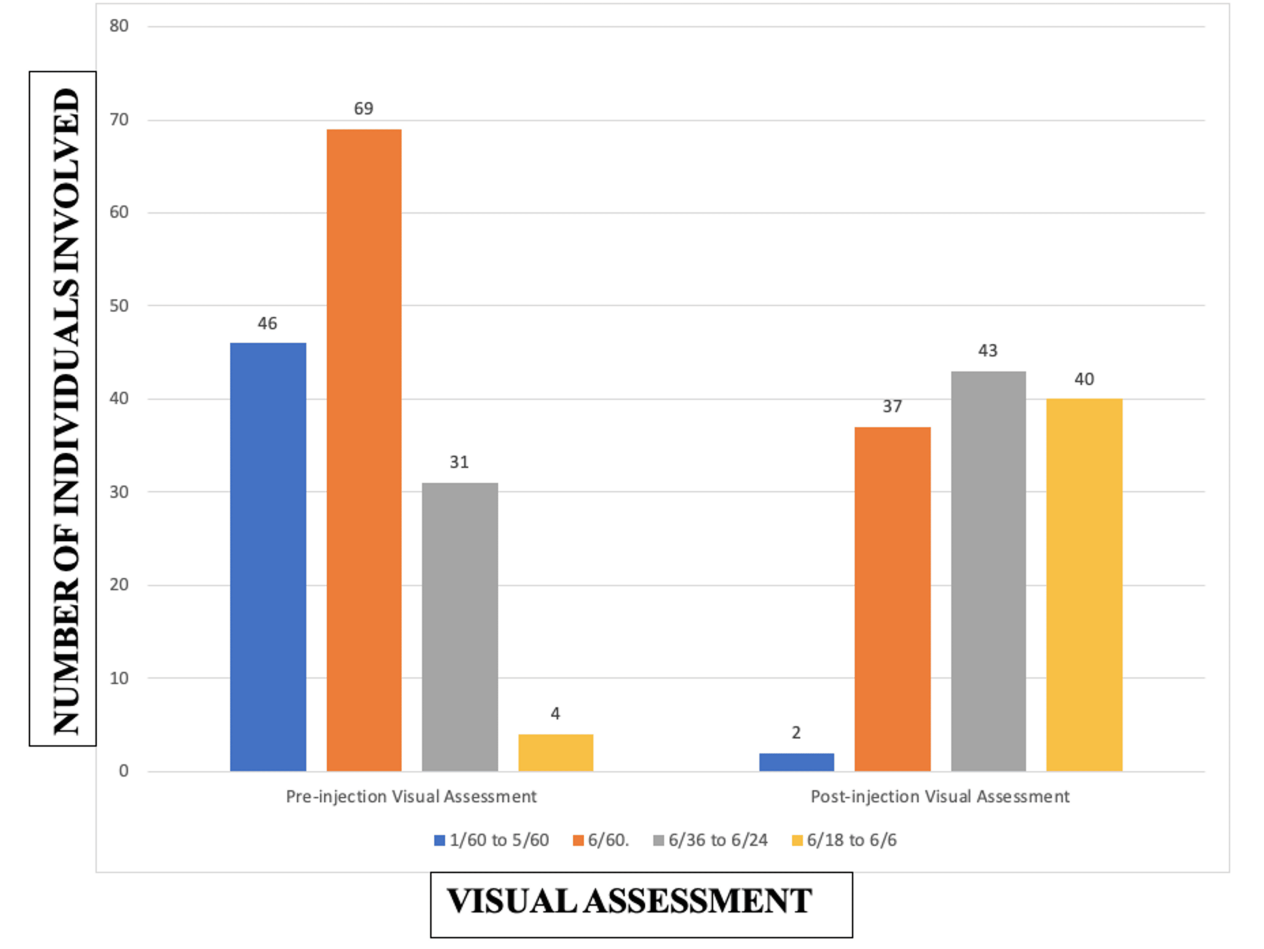
Pre and post-injection visual assessment in the study population. Data are of 122 individuals post-injection because 28 individuals had missed their appointments in the initial phase, i.e., 14 to 90 days, itself. Blue color: Individuals having a visual acuity between 1/60 and 5/60. Orange color: Individuals having a visual acuity of 6/60. Gray color: Individuals having a visual acuity between 6/36 and 6/24. Yellow color: Individuals having a visual acuity between 6/18 and 6/6.

Pre-injection, 30.7% (46 individuals) had a visual acuity of 1/60 to 5/60, 46% (69 individuals) had a visual acuity of 6/60, 20.7% (31 individuals) had a visual acuity of 6/36 to 6/24, and 2.7% (4 individuals) had a visual acuity of 6/18 to 6/6. Post-injection, 1.6% (2 individuals) had a visual acuity of 1/60 to 5/60, 30.3% (37 individuals) had a visual acuity of 6/60, 35.2% (43 individuals) had a visual acuity of 6/36 to 6/24, and 32.8% (40 individuals) had a visual acuity of 6/18 to 6/6 (Figure 2).

Through the McNemar test, there was a significant improvement (p < 0.05) in the vision of the patients following the treatment (Table 9).

**Table 9:** Visual acuity changes in the study population. McNemar test. Data are of 122 individuals post-injection because 28 individuals had missed their appointments in the initial phase, i.e., 14 to 90 days, itself.

Visual assessment			Post-injection visual assessment				P-value*
			1/60 to 5/60	6/60	6/36 to 6/24	6/18 to 6/6	
Pre-injection visual assessment	1/60 to 5/60	Frequency (Percent)	2 (4.5%)	35 (79.5%)	5 (11.4%)	2 (4.5%)	<0.001
	6/60	Frequency (Percent)	0 (0%)	2 (4.5%)	32 (72.7%)	10 (22.7%)	
	6/36 to 6/24	Frequency (Percent)	0 (0%)	0 (0%)	6 (20%)	24 (80%)	
	6/18 to 6/6	Frequency (Percent)	0 (0%)	0 (0%)	0 (0%)	4 (100%)	
Total		Frequency (Percent)	2 (1.6%)	37 (30.3%)	43 (35.2%)	40 (32.8%)	

Among the patients with mild to severe non-proliferative diabetic retinopathy, most patients showed improvement in their visual acuity from the baseline after injection (Table 10).

**Table 10:** Visual acuity changes of patients having mild to severe NPDR in the study population. Univariate analysis; p-value not indicated. Data are of 122 individuals post-injection because 28 individuals had missed their appointments in the initial phase, i.e., 14 to 90 days, itself. NPDR = non-proliferative diabetic retinopathy

Post-injection visual assessment			6/60	6/36 to 6/24	6/18 to 6/6	Total
Pre-injection visual assessment	1/60 to 5/60	Frequency (percent)	13 (92.9%)	1 (7.1%)	0 (0%)	14 (100%)
	6/60	Frequency (percent)	0 (0%)	5 (83.3%)	1 (16.7%)	6 (100%)
	6/36 to 6/24	Frequency (percent)	0 (0%)	0 (0%)	5 (100%)	5 (100%)
	6/18 to 6/6	Frequency (percent)	0 (0%)	0 (0%)	2 (100%)	2 (100%)
Total		Frequency (percent)	13 (48.1%)	6 (22.2%)	8 (29.6%)	27 (100%)

Among the patients with proliferative diabetic retinopathy, the majority of patients showed improvement in their visual acuity from the baseline after injection (Table 11).

**Table 11:** Visual acuity changes of patients having PDR in the study population. Univariate analysis; p-value not indicated. Data are of 122 individuals post-injection because 28 individuals had missed their appointments in the initial phase, i.e., 14 to 90 days, itself. PDR = proliferative diabetic retinopathy

Post-injection visual assessment			1/60 to 5/60	6/60	6/36 to 6/24	6/18 to 6/6	Total
Pre-injection visual assessment	1/60 to 5/60	Frequency (percent)	2 (14.3%)	10 (71.4%)	1 (7.1%)	1 (7.1%)	14 (100 %)
	6/60	Frequency (percent)	0 (0%)	2 (22.2%)	6 (66.7%)	1 (11.1%)	9 (100%)
	6/36 to 6/24	Frequency (percent)	0 (0%)	0 (0%)	1 (12.5%)	7 (87.5%)	8 (100%)
Total		Frequency (percent)	2 (6.5%)	12 (38.7%)	8 (25.8%)	9 (29.0%)	31 (100%)

Among the patients with nAMD, the majority of patients showed improvement in their visual acuity from the baseline after injection (Table 12).

**Table 12:** Visual acuity changes of patients having nAMD in the study population. Univariate analysis; p-value not indicated. Data are of 122 individuals post-injection because 28 individuals had missed their appointments in the initial phase, i.e., 14 to 90 days, itself. nAMD = neovascular age-related macular degeneration

Post-injection Visual assessment ->			6/60	6/36 to 6/24	6/18 to 6/6	Total
Pre-injection Visual Assessment	1/60 to 5/60	Frequency (Percent)	5 (83.3%)	0 (0%)	1 (16.7%)	6 (100%)
	6/60	Frequency (Percent)	0 (0%)	13 81.3%)	3 (18.8%)	16 (100%)
	6/36 to 6/24	Frequency (Percent)	0 (0%)	4 (28.6%)	10 (71.4%)	14 (100%)
	6/18 to 6/6	Frequency (Percent)	0 (0%)	0 (0%)	2 (100%)	2 (100%)
Total		Frequency (Percent)	5 (13.2%)	17 (44.7%)	16 (42.1%)	38 (100%)

Among the patients with RVO, the majority of patients showed improvement in their visual acuity from the baseline after injection (Table 13).

**Table 13:** Visual acuity changes of patients having RVO in the study population. Univariate analysis; p-value not indicated. Data are of 122 individuals post-injection because 28 individuals had missed their appointments in the initial phase, i.e., 14 to 90 days, itself. RVO = retinal vein occlusion

Post-injection visual assessment			6/60	6/36 to 6/24	6/18 to 6/6	Total
Pre-injection visual assessment	1/60 to 5/60	Frequency (percent)	7 (70%)	3 (30%)	0 (0%)	10 (100%)
	6/60	Frequency (percent)	0 (0%)	8 (61.5%)	5 (38.5%)	13 (100%)
	6/36 to 6/24	Frequency (percent)	0 (0%)	1 (33.3%)	2 (66.7%)	3 (100%)
Total		Frequency (percent)	7 (26.9%)	12 (46.2%)	7 (26.9%)	26 (100%)

Through the Wilcoxon signed-rank test, there was an overall significant reduction (p < 0.001) in the central subfield macular thickness, volume cube, and thickness average. cube after the treatment (Table 14).

**Table 14:** OCT parameters in the study population. Wilcoxon signed-rank test. ^a^: post-central macular thickness (μm) < pre-central macular thickness (μm). ^b^: post-central macular thickness (μm) > pre-central macular thickness (μm); ^c^: post-central macular thickness (μm) = pre-central macular thickness (μm); ^d^: post-volume cube (mm^3^) < pre-volume cube (mm^3^); ^e^: post-volume cube (mm^3^) > pre-volume cube (mm^3^); ^f^: post-volume cube (mm^3^) = pre-volume cube (mm^3^); ^g^: post-thickness average cube (μm) < pre-thickness average cube (μm); ^h^: post-thickness average cube (μm) > pre-thickness average cube (μm); ^i^:  post-thickness average cube (μm) = pre-thickness average cube (μm). OCT = optical coherence tomography

OCT parameters		N	Mean rank	Sum of ranks	P-value
Post-intravitreal injection central subfield macular thickness (μm) – pre-intravitreal injection central subfield macular thickness (μm)	Negative ranks	117^a^	63.07	7,379.00	<0.001
	Positive ranks	5^b^	24.80	124.00	
	Ties	0^c^			
	Total	122			
Post-intravitreal injection volume cube (mm^3^) – pre-intravitreal injection volume cube (mm^3^)	Negative ranks	91^d^	66.49	6,051.00	<0.001
	Positive ranks	31^e^	46.84	1,452.00	
	Ties	0^f^			
	Total	122			
Post-intravitreal injection thickness average cube (μm) – pre-intravitreal injection thickness average cube (μm)	Negative ranks	90^g^	64.11	5,770.00	<0.001
	Positive tanks	29^h^	47.24	1,370.00	
	Ties	3^i^			
	Total	122			

Through the Wilcoxon signed-rank test, there was a significant reduction (p < 0.05) in the central subfield macular thickness, volume cube, and thickness average cube after the treatment in all four groups of diagnosis (Table 15).

**Table 15:** OCT parameters in all four diagnostic groups of the study population. Wilcoxon signed-rank test. ^a^: post-central macular thickness (μm) < pre-central macular thickness (μm); ^b^: post-central macular thickness (μm) > pre-central macular thickness (μm); ^c^: post-central macular thickness (μm) = pre-central macular thickness (μm); ^d^: post-volume cube (mm^3^) < pre-volume cube (mm^3^); ^e^: post-volume cube (mm^3^) > pre-volume cube (mm^3^); ^f^: post-volume cube (mm^3^) = pre-volume cube (mm^3^); ^g^: post-thickness average cube (μm) < pre-thickness average cube (μm); ^h^: post-thickness average cube (μm) > pre-thickness average cube (μm); ^i^: post-thickness average cube (μm) = pre-thickness average cube (μm). NPDR = non-proliferative diabetic retinopathy; PDR = proliferative diabetic retinopathy; nAMD = neovascular age-related macular degeneration; RVO = retinal vein occlusion; OCT = optical coherence tomography

OCT parameters			N	Mean rank	Sum of ranks	P-value
Mild to severe NPDR	Post-intravitreal injection central subfield macular thickness (μm) – pre-intravitreal injection central subfield macular thickness (μm)	Negative ranks	27^a^	14.00	378.00	<0.001
		Positive ranks	0^b^	.00	.00	
		Ties	0^c^			
		Total	27			
	Post-intravitreal injection volume cube (mm^3^) – Pre-intravitreal injection volume cube (mm^3^)	Negative ranks	23^d^	13.52	311.00	0.003
		Positive ranks	4^e^	16.75	67.00	
		Ties	0^f^			
		Total	27			
	Post-intravitreal injection thickness average cube (μm) – Pre-intravitreal injection thickness average cube (μm)	Negative ranks	24^g^	13.75	330.00	0.001
		Positive ranks	3^h^	16.00	48.00	
		Ties	0^i^			
		Total	27			
PDR	Post-intravitreal injection central subfield macular thickness (μm) – pre-intravitreal injection central subfield macular thickness (μm)	Negative ranks	30^a^	16.43	493.00	<0.001
		Positive ranks	1^b^	3.00	3.00	
		Ties	0^c^			
		Total	31			
	Post-intravitreal injection volume cube (mm^3^) – pre-intravitreal injection volume cube (mm^3^)	Negative ranks	20^d^	19.30	386.00	0.007
		Positive ranks	11^e^	10.00	110.00	
		Ties	0^f^			
		Total	31			
	Post-intravitreal injection thickness average cube (μm) – pre-intravitreal injection thickness average cube (μm)	Negative ranks	21^g^	17.43	366.00	0.001
		Positive ranks	8^h^	8.63	69.00	
		Ties	2^i^			
		Total	31			
nAMD	Post-intravitreal injection central subfield macular thickness (μm) – pre-intravitreal injection central subfield macular thickness (μm)	Negative Ranks	35^a^	20.17	706.00	<0.001
		Positive Ranks	3^b^	11.67	35.00	
		Ties	0^c^			
		Total	38			
	Post-intravitreal injection volume cube (mm^3^) – pre-intravitreal injection volume cube (mm^3^)	Negative ranks	28^d^	20.50	574.00	0.003
		Positive ranks	10^e^	16.70	167.00	
		Ties	0^f^			
		Total	38			
	Post-intravitreal injection thickness average cube (μm) – pre-intravitreal injection thickness average cube (μm)	Negative ranks	25^g^	20.92	523.00	0.010
		Positive ranks	12^h^	15.00	180.00	
		Ties	1^i^			
		Total	38			
RVO	Post-intravitreal injection central subfield macular thickness (μm) – pre-intravitreal injection central subfield macular thickness (μm)	Negative ranks	25^a^	13.86	346.50	<0.001
		Positive ranks	1^b^	4.50	4.50	
		Ties	0^c^			
		Total	26			
	Post-intravitreal injection volume cube (mm^3^) – pre-intravitreal injection volume cube (mm^3^)	Negative ranks	20^d^	15.25	305.00	0.001
		Positive ranks	6^e^	7.67	46.00	
		Ties	0^f^			
		Total	26			
	Post-intravitreal injection thickness average cube (μm) – pre-intravitreal injection thickness average cube (μm)	Negative ranks	20^g^	13.75	275.00	0.011
		Positive ranks	6^h^	12.67	76.00	
		Ties	0^i^			
		Total	26			

## Discussion

A total of 150 consecutive eyes of patients with either DR, RVO, or nAMD treated with intravitreal bevacizumab were studied. Non-compliance or loss to follow-up is a major concern in India. We, therefore, studied factors influencing compliance in patients with these diseases undergoing intravitreal injection of bevacizumab.

Demography

In our study, the mean age of the patients was 59.51 years, the mean duration of diabetes was 9.79 years, the mean HbA1C level was 8.1, the mean duration of hypertension was 4.19 years, the mean pre-intravitreal injection central subfield macular thickness and post-intravitreal injection central subfield macular thickness were 393.87 and 301.42, respectively, the mean pre-intravitreal injection volume and post-intravitreal injection volume were 11.33 mm^3^ and 10.27 mm^3^, respectively, and the mean pre-intravitreal injection thickness and post-intravitreal injection thickness were 313.2 mm^3^ and 285.59 mm^3^, respectively (Table 1). This suggests significant improvement in the indices studied (p < 0.05) after they were given intravitreal injection. Kumar et al. described visual acuity response in patients with diffuse diabetic macular edema who were administered intravitreal bevacizumab [8]. All patients had significant macular edema with poor visual acuity following previous laser photocoagulation. All patients were given two bevacizumab injections six weeks apart. Following the intervention, improvement of vision was seen and macular thickness dropped by 123 µm.

In our study, we had a comparatively younger population with the mean age of the study population being 59.5 ± 10 years. Study participants were mainly businessmen or company workers and were graduates. Kelkar et al. in a retrospective study assessed the compliance and follow-up rates of RVO, AMD, and diabetic macular edema patients to treatment with intravitreal bevacizumab and ranibizumab. Of the 648 patients included in their study, two-thirds were males with an average age of 66.4 years, and the majority of the study participants were either farmers or housewives [7].

In this study, the majority of the individuals were from urban areas, while in a study by Abu-Yaghi et al. on factors affecting compliance to anti-VEGF treatment for diabetic macular edema in a cohort of Jordanian patients, patients included in the study were uneducated, retired, and from rural areas [9].

nAMD was present in 32% of the patients studied, followed by NPDR (25%), PDR (23%), and RVO (19%) (Table 2). Moreover, 69.3% were smokers. In the study by Kelkar et al., 36% had diabetic macular edema, 33.8% had AMD, and 30.1% were diagnosed with RVO. The burden of nAMD was similar and RVO was less when the two studies were compared and 75% of the individuals in their study were smokers [7].

Systemic illness

Overall, 62% of the individuals studied had diabetes mellitus. Among the diabetics studied, 54% were on medications. The average duration of diabetes in the patients in this study was 9.7 years. The mean HbA1c level was 8.1. Of the patients studied, 65% had hypertension and 85% were on medications for hypertension. Further, 45% of individuals had a positive family history of diabetes mellitus and hypertension (Table 3), with 51% of individuals having other comorbidities. In a retrospective study by Kelkar et al., 60% were diabetics with 50% on medications, and 58% were hypertensives with 75% on medications [7].

Compliance factors

Non-compliance to treatment or loss to follow-up is a major concern during the treatment of patients. We, therefore, evaluated the factors affecting compliance in patients with DR, RVO, and AMD. We also evaluated the effect of gender, cost of treatment, and type of disease present for which the patients underwent intravitreal injection.

Non-compliance rate in our study was 21% (Table 4), while in the study by Kelkar et al., non-compliance to therapy was seen in 51.5% of patients studied [7]. The higher non-compliance rates in their study could be due to the cost of therapy and poor awareness of the effect of treatment. In this study, over 90% of the individuals were willing to get treatment. All patients were well-informed about their disease. Of the patients studied, 89% were worried about the visual outcome. Over half of the patients (53%) were worried about the side effects of medications, and two-thirds were afraid of getting injected. Over 90% had disbelief in the treatment. Further, 13% had experienced a lack of support from the family for treatment. Over half of the patients had transportation issues, and all patients in our study were covered by governmental health schemes (Table 5).

Shahzad et al. evaluated the non-adherence to anti-VEGF injections. The non-adherence ranged from 17.5 to 35% in their study. Age, unhappiness with treatment outcomes, financial strain, older age/comorbidities, travel distance/social isolation, dissatisfaction, and discomfort/pain were found to be the reasons for non-adherence. Another study reported non-adherence ranging from 51.6 to 68.8%. This was attributed to travel issues and COVID-19 exposure anxiety [10].

In this study, 65% of patients had taken one anti-VEGF injection, 20% had taken two injections, and the rest had taken more than two (Table 6). Overall, 80% of patients were compliant with treatment. The compliance was higher than the findings of Kelkar et al. at 45.22%. This was probably because all our patients were beneficiaries of government health schemes which reduced the cost of their treatment [7].

Ramakrishnan et al. studied the correlation of adherence with visual acuity in nAMD patients. They found that 17.7% of patients did not attend over two continuous months. In this study, the missed visit which was considered on 14 to 90 days was 18.7%, and non-follow-up for more than one year was 30% (Figure 1) [11].

We also compared compliance to treatment and diagnosis (Table 7). Compliance was 65.8% and 85.7% in the NPDR and PDR groups, respectively, 79.2% in the nAMD group, and 89.7% in the RVO group. This was, however, not clinically significant (p = 0.073). Kelkar et al. in their study had 34.8% with NPDR and PDR, respectively, 34.5% with nAMD, and 30.7% with RVO. They also found no clinically significant association between the disease for which they were getting intravitreal injections [7].

In our study, we compared compliance to treatment and gender. Compliance with treatment was 82.8% in males while it was 74.6% in females, but this difference was not clinically significant (p = 0.223). In the study by Kelkar et al., they also noticed lower compliance in females compared to males [7]. We also studied compliance considering treatment and occupation. We found that compliance to treatment in the business group was 78.6%; in company workers, it was 78.9%; in housewives, it was 75.7%; in farmers, it was 72.7%; and in teachers, it was 90.9%. The results were not significant clinically (p = 0.662). We studied compliance to treatment and educational status. Compliance with treatment was 87.5%, 80%, and 71% among 10th-pass individuals, graduates, and uneducated individuals, respectively. The results were not significant clinically (p = 0.329). We also compared compliance to treatment and employment status, with 85.2% in employed participants, 70% in farmers, 80% in retired individuals, and 76.3% in unemployed individuals. The results were not significant clinically (p = 0.717). The study by Abu-Yaghi et al. also found similar results [9]. We compared compliance to treatment and whether the patient was from urban/rural areas. Compliance with treatment in urban areas was 79.5%, while in patients from rural areas, it was 78.9%. The results were not clinically significant (p = 0.946).

Polat et al. studied patients in Turkey undergoing anti-VEGF injections [12]. The patients had to pay a proportion of the price for anti-VEGF injections despite health insurance. They found non-adherence to be 8.3%. In our study, we compared compliance to treatment and financial status. We found compliance to treatment in patients with a good financial status at 78.6% while in those with poor financial status at 81.6% (p = 0.692). This is probably because all our patients were beneficiaries of government schemes that reduced the cost of their treatment. We compared compliance to take treatment and whether the patient was a smoker or not. Compliance to treatment in smokers was 76% but it was not clinically significant (p = 0.125). We compared compliance to treatment and whether the patient was a diabetic. Treatment compliance in a diabetic was 75.3%. The results were not clinically significant (p = 0.116). In the study by Kelkar et al., 60.1% of diabetic patients were compliant with treatment. We compared compliance to treatment and whether the patient was hypertensive. Compliance with treatment in a hypertensive was 78.4%. The results were not clinically significant (p = 0.688). In the study by Kelkar et al., 58% of hypertensive patients were compliant with treatment [7].

Through the Mann-Whitney U test, we found out that younger patients with a shorter duration of diabetes had clinically significant results (p = 0.009) with higher compliance rates (Table 8). Kelkar et al. observed a significant association between the female gender and loss of follow-up to anti-VEGF therapy. Previous history of diabetes and hypertension were also significantly associated with loss of follow‐up to anti‐VEGF therapy. However, in our study, younger patients (p = 0.032) with a shorter duration of diabetes mellitus (p = 0.009) had significantly higher compliance rates [7].

Clinical characteristics

In our study, 30.7% (46 individuals) had a visual acuity of 1/60 to 5/60, 46% (69 individuals) had a visual acuity of 6/60, 20.7% (31 individuals) had a visual acuity of 6/36 to 6/24, and 2.7% (4 individuals) had a visual acuity of 6/18 to 6/6. Post-injection, 1.6% (2 individuals) had a visual acuity of 1/60 to 5/60, 30.3% (37 individuals) had a visual acuity of 6/60, 35.2% (43 individuals) had a visual acuity of 6/36 to 6/24, and 32.8% (40 individuals) had a visual acuity of 6/18 to 6/6 (Figure 2). There was a significant reduction in the central macular thickness, volume cube, and thickness average cube after the treatment (p < 0.01). There was a significant improvement in the vision of the patients following the treatment which was clinically significant (p < 0.001).

Visual assessment was done pre-injection and post-injection in our study (Table 9). There was a significant improvement in the vision of DR patients (Tables 10, 11), nAMD (Table 12), and RVO (Table 13), following the treatment, as analyzed by the McNemar test. In an interventional study to evaluate the efficacy and safety of intravitreal bevacizumab injections for the treatment of nAMD (that received either intravitreal bevacizumab, photodynamic therapy with verteporfin or intravitreal pegaptanib intravitreal injections or sham injections), 32% of patients in the bevacizumab group improved their baseline visual acuity, while in the present study, visual acuity (6/18 to 6/6) in the pre-intervention group improved from 3% to 33% which was clinically significant (p < 0.001). Intravitreal bevacizumab was effective in improving the visual acuity (odds ratio: 18:1) over the control.

Kook et al. assessed the long-term effectiveness of bevacizumab in diabetic macular edema patients. At the six and 12-month follow-ups, 47-48% of patients had received at least three intravitreal bevacizumab injections. The study resulted in long-term reduction (p < 0.05) in central retinal thickness, even in patients with chronic diabetes with repeated injections. In these individuals, the average increase in visual acuity was correlated with a mean reduction in retinal thickness. However, in this study, we included both macular degeneration as well as RVO which showed significant improvement in visual acuity (p < 0.001) as well as a reduction in central retinal thickness (p < 0.05) [13].

Sacu et al. studied the effect of intravitreal bevacizumab and photodynamic treatment with intravitreal triamcinolone in patients with AMD. Visual acuity was observed to be improved. During 12 months, the intravitreal bevacizumab group documented a superior visual acuity result compared to the photodynamic treatment group. We compared the results of patients who received intravitreal bevacizumab injections before and after the intervention in patients with DR, AMD, and RVO and documented a significant improvement in vision (Table 9) as well as a reduction in central macular thickness (Table 14) which was clinically significant (p < 0.001) [14].

Arevalo et al., in a retrospective analysis, investigated the impact of intravitreal bevacizumab on PDR for retinal neovascularization [15]. They found that intravitreal bevacizumab resolved retinal neovascularisation in patients with PDR and prior PRP. Similarly, in our study, there was a significant improvement (p < 0.001) in visual acuity and central macular thickness of PDR patients receiving intravitreal bevacizumab injection (Table 15). Similar observations in visual acuity improvement with intravitreal bevacizumab were observed by Wirkkala et al. (p < 0.05) and Hall et al. (p < 0.001) [16].

Limitations

The validity of our findings is limited as the study was limited to a single hospital and a single surgeon. Due to the lack of a control group, we could not compare the risk factors and outcomes between the two groups. The sample size was small. Reasons for loss to follow-up were not available, though effort was made to contact patients to find the reason. Our results were only based on the use of bevacizumab anti-VEGF injection. We did not take into consideration other anti-VEGF agents such as ranibizumab and aflibercept.

## Conclusions

This study demonstrated intravitreal bevacizumab injection as an effective and promising treatment approach for patients with DR, RVO, and nAMD and factors affecting compliance which play an important role in the visual outcomes of the patients. In our study, four-fifths of the patients (79.3%) were compliant with treatment and visual improvement was significant. In addition, there was a significant reduction in the macular thickness of DR, RVO, and AMD patients after treatment. Factors for non-compliance included in our study were the need for follow-up, educational and employment status, diabetic or hypertensive profile, other comorbidities, afraid of getting injected and its side effects, transportation issues, financial burden, and treatment disbelief. Younger patients and those with a shorter duration of diabetes had significantly higher compliance. We recommend that similar randomized control trials and multi-centric studies should be conducted to confirm the results of our study.

For future studies on intravitreal anti-VEGF therapies, several recommendations can help advance the field and optimize patient outcomes, including focusing on long-term safety and efficacy, evaluating extended-release formulations, exploring combination therapies, personalizing treatment approaches, advancing drug delivery systems, incorporating advanced imaging techniques, investigating economic and access factors, and promoting collaborative research.
